# Pandemic extra-intestinal pathogenic *Escherichia coli* (ExPEC) clonal group O6-B2-ST73 as a cause of avian colibacillosis in Brazil

**DOI:** 10.1371/journal.pone.0178970

**Published:** 2017-06-08

**Authors:** Marcos Paulo Vieira Cunha, Andre Becker Saidenberg, Andrea Micke Moreno, Antonio José Piantino Ferreira, Mônica Aparecida Midolli Vieira, Tânia Aparecida Tardelli Gomes, Terezinha Knöbl

**Affiliations:** 1 Department of Pathology, Faculdade de Medicina Veterinária e Zootecnia, Universidade de São Paulo, São Paulo, Brazil; 2 Department of Preventive Veterinary Medicine, Faculdade de Medicina Veterinária e Zootecnia, Universidade de São Paulo, São Paulo, Brazil; 3 Department of Microbiology, Immunology and Parasitology, Universidade Federal de São Paulo, Escola Paulista de Medicina, São Paulo, Brazil; Beijing Institute of Microbiology and Epidemiology, CHINA

## Abstract

Extra-intestinal pathogenic *Escherichia coli* (ExPEC) represent an emerging pathogen, with pandemic strains increasingly involved in cases of urinary tract infections (UTIs), bacteremia, and meningitis. In addition to affecting humans, the avian pathotype of ExPEC, avian pathogenic *E*. *coli* (APEC), causes severe economic losses to the poultry industry. Several studies have revealed overlapping characteristics between APEC and human ExPEC, leading to the hypothesis of a zoonotic potential of poultry strains. However, the description of certain important pandemic clones, such as Sequence Type 73 (ST73), has not been reported in food sources. We characterized 27 temporally matched APEC strains from diverse poultry farms in Brazil belonging to the O6 serogroup because this serogroup is frequently described as a causal factor in UTI and septicemia in humans in Brazil and worldwide. The isolates were genotypically characterized by identifying ExPEC virulence factors, phylogenetically tested by phylogrouping and multilocus sequence type (MLST) analysis, and compared to determine their similarity employing the pulsed field gel electrophoresis (PFGE) technique. The strains harbored a large number of virulence determinants that are commonly described in uropathogenic *E*. *coli* (UPEC) and sepsis associated *E*. *coli* (SEPEC) strains and, to a lesser extent in neonatal meningitis associated *E*. *coli* (NMEC), such as *pap* (85%), *sfa* (100%), *usp* (100%), *cnf*1 (22%), *kps*MTII (66%), *hly*A (52%), and *ibe*A (4%). These isolates also yielded a low prevalence of some genes that are frequently described in APEC, such as *iss* (37%), *tsh*, *omp*T, and *hly*F (8% each), and *cvi/cva* (0%). All strains were classified as part of the B2 phylogroup and sequence type 73 (ST73), with a cluster of 25 strains showing a clonal profile by PFGE. These results further suggest the zoonotic potential of some APEC clonal lineages and their possible role in the epidemiology of human ExPEC, in addition to providing the first description of the O6-B2-ST73 clonal group in poultry.

## Introduction

Extra-intestinal pathogenic *Escherichia coli* (ExPEC) are commonly isolated pathogens from a wide variety of diseases in animals and humans. In recent decades, ExPEC has become an emerging disease that is responsible for increasing economic and health burdens on society, with pandemic strains involved in community setting and healthcare-associated outbreaks [1].

*E*. *coli* is now a leading cause of urinary tract infections (UTIs), meningitis, and bacteremia, and it is responsible for high morbidity and mortality rates that surpass those associated with infections caused by intestinal pathogenic *E*. *coli* pathotypes such as Shiga-toxin producing *E*. *coli* (STEC) related to hemolytic uremic syndrome [1, 2].

The ExPEC pathotype is subdivided into UPEC (uropathogenic *E*. *coli*), NMEC (neonatal meningitis associated *E*. *coli*), sepsis associated *E*. *coli* (SEPEC), and APEC (avian pathogenic *E*. *coli*). ExPEC can be classified using various genotyping methods, such as the detection of virulence determinants encoded by genes that allow the bacteria to adhere, invade, acquire iron, and evade the host immune response. Other techniques include phylogrouping, in which most ExPEC belong to the predominant phylogroup B2 and, to a reduced degree, to phylotype D, and the multilocus sequence typing (MLST), which allows the assignment of closely related strains in clonal groups or complexes as a sequence type (ST). These standardized classifications have facilitated the identification and surveillance of pandemic strains that cause nosocomial and community outbreaks [2, 3]. Multidrug resistant (MDR) strains are common, with increasing frequencies of resistance being reported over the decades. This phenomenon, is of extreme concern regarding remaining antibiotic treatment options [4].

Subgroup APEC is responsible for severe economic losses to the poultry industry worldwide as a cause of airsacullitis, polyserositis, septicemia, poor growth performance and carcass condemnation in affected flocks, hindering the production of an important low cost meat source in addition to affecting the welfare of farmed poultry [5, 6].

The main serogroups involved in APEC include O1, O2 and O78. In addition, several clones circulating among poultry affected by colibacillosis and isolated in poultry retail products (retail pathogenic *E*. *coli*–RPEC) have been described as sharing overlapping characteristics with human ExpEC strains in terms of serogroups, virulence genes, phylogroups, and STs. Furthermore, indistinguishable or closely related clones to those causing disease in humans have been identified by methods such as pulsed field gel electrophoresis (PFGE) [7–10].

Genomic studies have further reinforced the finding that some APEC are highly similar to UPEC and NMEC. In addition, *in vivo* inoculation of APEC and RPEC strains in mammalian experimental models was able to replicate the disease, as well as by inoculating human derived isolates in chickens and turkeys, thus demonstrating the non host-specificity of some ExPEC strains [11–14].

The growing evidence of reports showing that a subset of *E*. *coli* from poultry have zoonotic potential has led to the hypothesis that the route of transmission of ExPEC to humans occurs via the consumption of food from an animal origin, especially retail poultry products, leading to the description of strains implicated in urinary tract infections as food-borne urinary tract infections (FUTI) [15].

Pandemic ExPEC lineages have been described as a cause of disease in humans and livestock, including the B2-ST131, B2-ST95 and D-ST69 clones [2, 3]. The B2-ST73 lineage is a well-known high-risk clonal group that is associated worldwide with nosocomial and community-acquired human infections such as UTI and sepsis [1, 16, 17].

The aim of this study was to characterize a number of APEC O6-B2-ST73 strains isolated from colibacillosis in poultry from Brazil. To the best of our knowledge, the ST73 clonal group has not been described as a cause of avian colibacillosis, and we further discuss the characteristics of the isolates and their similarities to human ExPEC.

## Materials and methods

This study was approved by the Ethics Committee of São Paulo University and authorized for scientific purposes (CEUA 1840110416), and employed a previous collection of *E*. *coli* strains from temporally matched but geographically diverse outbreak cases of colibacillosis. These outbreaks affected several broiler farms located in four different states in Southern and Southeastern Brazil and the strains were deposited in the bacterial collection of the Avian Medicine Laboratory of the University of Sao Paulo, Brazil.

Samples were collected from symptomatically recently euthanized birds by aseptically dissecting specimens and observing fibrinous lesions suggestive of colisepticemia (perihepatitis or omphalitis) in all necropsied cases. Swabs of these lesions were obtained, cultured in brain and heart infusion broth and subcultured on MacConkey agar plates. Pure growth *E*. *coli* cultures were selected for serotyping according to previously described methods [18], and stored at -80°C in 20% glycerol/Luria-Bertani medium. Twenty-seven strains from this collection, identified serologically as serogroup O6, were selected for further characterization due to the infrequent finding of this serotype among APEC strains, and its possible zoonotic potential linked to clones sharing the same O6 classic human UPEC serotype [19].

### Virulence determinant testing

The strains were tested by PCR, as previously reported for the presence of virulence genes encoding several adhesins (*fim*H, *crl*, *pap*C, *sfa*, *tsh*, *afa*), toxins (*vat*, *ast*A, *hly*A, *sat*, *cnf*), protectins/serum resistance (*cvi/cva*, *iss*, *kps*MTII, *omp*T), iron acquisition/uptake systems (*iro*N, *iuc*D, *iut*A, *irp*2, *fyu*A), pathogenicity island markers (PAI I_CFT073_—*malX*, PAI II_CFT073_, PAI I_536_, PAI II_536_) and other virulence traits (*ibe*A, *usp*, *hly*F) that are shared among APEC, UPEC and NMEC [7, 20–23]. The primer references for each tested gene are listed in S1 Table. Strains from a previous study were included as positive controls [24]. The *Escherichia coli* K12 strain served as a negative control for the reactions.

### Phylogrouping, PFGE, and MLST typing

Phylogenetic analysis was performed using the improved phylotyping method reported by Clermont et al. (2013) to classify the isolates in one of the eight phylogenetic groups [25]. The strains were also subjected to MLST according to the Achtman scheme (http://mlst.ucc.ie/mlst/mlst/dbs/Ecoli) and employing the described primers and protocols.

PFGE was performed using the restriction enzyme *Xba*I according to previously described methods and following the protocol of CDC PulseNet (www.cdc.gov/pulsenet) [26]. The PFGE results were analyzed with Bionumerics 7.5 software (Applied Maths NV, Saint-Martens-Latem, Belgium). The similarities between strains were calculated using the Dice coefficient with an optimization of 1%. The dendrograms were obtained employing the Unweighted Pair Group Method with Arithmetic Average (UPMGA) clustering algorithm. The strains were considered belonging to different pulsotypes when differing by four or more bands in their restriction profiles.

### Antimicrobial susceptibility testing

Antimicrobial resistance was assessed by the disk diffusion method according to the CLSI protocol [27]. The following antibiotics were tested: ampicillin, cefotaxime, ciprofloxacin, chloramphenicol, streptomycin, amikacin, gentamicin, tetracycline, trimethoprim-sulfamethoxazole, and sulfonamides. *E*. *coli* strain ATCC 25922 was used for quality control.

## Results

### Virulence determinants testing, phylogrouping, PFGE and MLST typing

**Fig 1:**
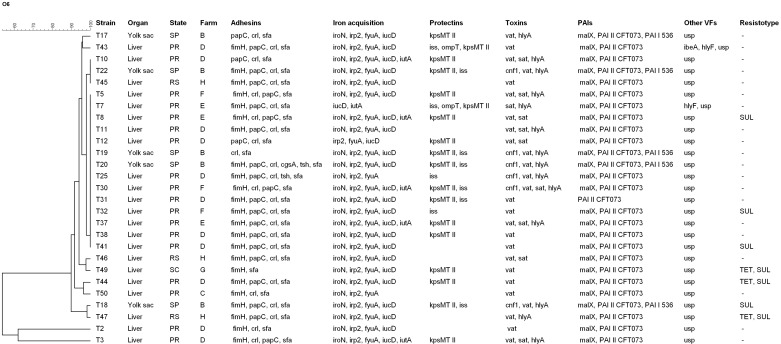
XbaI-PFGE dendrogram generated with the Bionumerics software based on the Dice similarity index indicating the genetic relatedness of 27 ExPEC O6-B2-ST73 strains from poultry. **The level of similarity (%) is shown at the top**. A, B and C indicate the three pulsotypes. States: PR = Paraná, RS = Rio Grande do Sul, SC = Santa Catarina, SP = São Paulo. VFs = Virulence factor, SUL = Sulfonamides, TET = Tetracycline.

All 27 strains showed the same Sequence Type 73 (ST73) by MLST, phylogenetic group B2, and the presence of genes encoding virulence factors that are found among ExPEC isolates (Table 1, Fig 1). The results revealed a relatively low prevalence or complete absence of genes that are frequently found in APEC, such as *tsh*, *cvi/cva*, *omp*T, *iss*, and *hly*F. Concomitantly, these strains also exhibited positivity for genes that are more commonly described in human UPEC/SEPEC and NMEC strains, such as *hly*A, *cnf*1, *kps*MTII, *sat*, *usp*, *pap*C, *sfa*, and *ibe*A. [2, 7, 21].

**Table 1:** Prevalence of virulence markers in 27 strains of APEC O6-B2-ST73.

Virulence gene	Description	*N* (%)
*fim*H	Type I fimbriae	23 (85)
*pap*C	P fimbriae	23 (85)
*crl*	Regulatory gene of curli	26 (96)
*sfa*	S fimbriae	27 (100)
*tsh*	Temperature-sensitive hemagglutinin	2 (8)
*afa*BC	Afimbrial adhesin	0 (0)
*iro*N	Catecholate siderophore (salmochelin) receptor	25 (92)
*irp2*	Iron-repressible protein (yersiniabactin synthesis)	26 (96)
*fyu*A	Ferric yersinia uptake (yersiniabactin receptor)	26 (96)
*iut*A	Ferric aerobactin receptor	6 (22)
*iuc*D	Aerobactin synthesis	25 (92)
*kps*MT II	Group II capsule antigens	18 (66)
*iss*	Increased serum survival	10 (37)
*omp*T	Outer membrane protease gene	2 (8)
*cvi/cva*	Colicin V (ColV)	0 (0)
*vat*	Vacuolating autotransporter toxin	26 (96)
*sat*	Secreted autotransporter toxin	10 (37)
*hly*A	Hemolysin A	14 (52)
*cnf*1	Cytotoxic necrotizing factor 1	6 (22)
*ast*A	EAST1 (heat-stable cytotoxin)	0 (0)
*usp*	Uropathogenic specific protein	27 (100)
*ibe*A	Invasion of brain endothelium	1 (4)
*hly*F	Avian Hemolysin	2 (8)
*malX* (PAI I_CFT073_)	Pathogenicity island I marker of UPEC CFT073	26 (96)
PAI II_CFT073_	Pathogenicity island II marker of UPEC CFT073	27 (100)
PAI I_536_	Pathogenicity island I marker of UPEC 536	5 (18)
PAI II_536_	Pathogenicity island II marker of UPEC 536	0 (0)

High frequencies of detection were observed for the *fim*H, *pap*C, *crl*, *sfa*, *iro*N, *irp*2, *fyu*A, *iuc*D, *kps*MTII, *vat*, *hly*A, and *usp* genes. Lower frequencies were reported for the *tsh*, *iut*A, *iss*, *omp*T, *sat*, *cnf*1, *ibe*A, and *hly*F genes. None of the strains appeared to harbor the *afa*BC, *cvi/cva*, or *ast*A genes (Table 1).

Pathogenicity islands were observed in 96% of the strains for PAI I_CFT073_—*malX*, 100% for PAI II_CFT073_, and 18% for PAI I_536_, while no strains were positive for PAI II_536_.

A total of 21 non-shared virulence profiles were detected. Common patterns consisting of 12 different genes were shared among three groups (two strains per group). These shared virulence groups yielded a profile consisting of *fim*H, *pap*C, *crl*, *sfa*, *iro*N, *irp*2, *fyu*A, *iuc*D, *vat*, *malX*, PAI II_CFT073_ and *usp*, with additional gene combinations differing between these strains (Fig 1).

The 27 strains were grouped into three pulsotypes by PFGE. In one pulsotype (A), 25 strains showed similarities >90% to 100%, which included strains from three different states (Fig 1). In contrast, groups B and C resulted in a similarity >60% with strains originating from the same state and farm. The overall geographical connection with regard to the similarity of DNA fragments varied among most strains and generally showed varying distributions per sampled state, in addition an assorted allocation among unrelated farms. A more common origin was primarily observed in clade A, in which several clones were detected on farms in the state of Paraná (PR) and identified as D and E (100% similarity).

### Antimicrobial susceptibility testing

An overall low resistance pattern was observed for all strains, with the majority exhibiting susceptibility to all tested drugs. Resistance to sulfonamides and tetracycline was observed in three strains, while resistance to sulfonamides alone was observed in four isolates (Fig 1).

## Discussion

The hypothesis that poultry are a source of ExPEC infections in humans has been the subject of study by several research groups that have provided ongoing descriptions of phenotypic, genotypic, and genomic similarities between avian sources of *E*. *coli* and human ExPEC strains [4]. This theory is linked to the challenging definition of the APEC pathotype itself due to the inherent genome plasticity of *E*. *coli*, which allows the frequent exchange, loss and acquisition of genetic materials located on mobile elements. Diverse APEC strains present varying sets of virulence encoding genes, which are capable of causing disease [28].

Nevertheless, several studies have shown that APEC strains can be mostly identified by the possession of some virulence genes that are not commonly found in human ExPEC isolates like those present on the colicin V plasmid [2, 29]. Despite this classification, a considerable overlap of virulence determinants with human ExPEC can be found among a subset of APEC, thus suggesting non host-specificity of these strains and underscoring their zoonotic potential [7, 30].

In our study, we found a surprisingly lower prevalence of virulence genes that are more commonly described in APEC while also reporting several genes that are more prevalent in UPEC and SEPEC strains and, to a reduced degree, in NMEC [2]. Of interest, the *iss*, *tsh*, *omp*T, *hly*F and *cvi/cva* genes are often present on large plasmids in APEC [7, 20]; however, in the current study *iss* was detected in 37% of the strains; *tsh*, *omp*T, and *hly*F in 8% each, and no positive results were obtained for the *cvi/cva* genes.

Several reports comparing the genotypic profiles of ExPEC isolates have described higher frequencies of these genes in APEC, albeit in very diverse percentages given the genetic varieties among APEC and other ExPEC pathotypes [7, 20, 30, 31]. For instance, in the USA, plasmid associated genes were described in >60% of a diverse population of APEC in comparison to UPEC strains, although considerable overlap was observed among a subset of avian isolates and those originating from human UTIs [31]. In addition, studies with APEC in Europe have also established that a significant difference among APEC in comparison to UPEC and NMEC was related to genes that are frequently found on plasmids [7, 9].

A comparison of our findings with other studies examining APEC to date is also complicated by the fact that few have focused on O6 APEC strains, and that the genetic backbone of the serogroup plays a role in the genotypic similarities and differences that may be detected. This phenomenon is particularly important in comparisons of O6 serogroup strains that are commonly reported to cause UTIs/septicemia [19]. Therefore it is interesting to note how the strains in the current study identified genotypic associations that are frequently described in human ExPEC.

For instance, we detected a large number of strains that were positive for genes encoding P and S fimbriae (*pap* 85%, *sfa* 100%), uropathogenic-specific protein (*usp* 100%), group II capsule antigens (*kps*MTII 66%), alpha hemolysin (*hly*A 52%), and various iron uptake systems. Additionally, a considerable number of strains were also positive for cytotoxic necrotizing factor (*cnf*1 22%) (Table 1). These virulence factors have been positively associated with strains isolated from humans presenting urinary infections or sepsis and in the specific case of the *hly*A, this gene has rarely been reported in APEC [2, 32].

Rodriguez-Siek et al. (2005), described the virulence genes detected in 524 APEC in comparison to 200 UPEC of assorted serogroups. The *pap* gene was found in 38.7% of the APEC and in 51.5% of the UPEC strains, while *sfa* was detected in 4.2% of the APEC and 31.5% of the UPEC strains. The *cnf*1 gene was present in 1.1% of the APEC and 27.5% of the UPEC strains. *kps*MTII was described in 24.8% of APEC whereas UPEC harbored this gene in 77.5% of the strains. *hly*A was reported only in 0.8% of APEC although it was observed in 31% of UPEC [31]. While in Brazil, studies have compared several APEC and ExPEC isolates and did not report *cnf*1 and *hly*A in any avian strains [10, 33].

The invasion brain endothelium gene (*ibe*A) is a virulence factor that is well recognized among meningitis-causing *E*. *coli* strains and is also an important factor contributing to higher levels of virulence in APEC [34]. Here, we report that 4% of the tested isolates were positive for the *ibe*A gene. A number of studies compared the prevalence of NMEC-related genes among APEC strains and UPEC observing overlaps between the pathotypes [10, 30, 33]. Ewers et al. (2007), described *ibe*A in 26.2% of APEC which suggested that APEC could be a source for other ExPEC of plasmid and chromosomally located genes [7].

Several pathogenicity islands have been reported in our strains, with a predominance of PAI II_CFT073_ found in all isolates, followed by PAI (malX) (96%) and PAI_536_ (18%), while PAI II_536_ was not detected in any strains. These PAIs are closely connected to UPEC pathogenicity by harboring several virulence genes [22]. The PAI I_CFT073_ has been reported among a select subset population of APEC isolates, as well as in other ExPEC pathotypes [10, 30]. These findings suggest extensive horizontal genetic exchange between some avian and human strains [7].

A wide variety of phylogroups have been detected among APEC isolates around but virulent strains generally tend to be associated with phylogroups B2 and D [24, 35]. The Sequence Type 73 is exclusively associated with the B2 phylogroup and has also been reported among O6 serogroup strains [19, 36]. All the present strains were classified as B2-ST73 according to the phylogrouping and MLST analysis.

One of the most common ExPEC isolated from humans around the world is the B2-ST73 *E*. *coli* clone, which includes the prototype strain CFT073, isolated from a human urosepsis case [1]. In the USA, the ST73 has been reported as the third most common ST among bacteremia cases, and in Europe, it accounts for one of the main sequence types described [17, 37]. In Brazil, ST73 isolates from humans were described in association with community outbreaks of UTIs in women, where they were the second most commonly reported sequence type and the predominant cause of UTIs in men [38, 39].

A number of sequence types are shared among poultry and human ExPEC strains, including dominant pandemic clones that have also been reported in cases of colibacillosis in Brazil, [4, 10, 33].The O6-B2-ST73 clone had not been described in poultry isolates to date, and thus this finding highlights the description of an ST that was up to now considered to be an adapted strain in humans without a recognized food source background [1, 4, 16].

Although this is the first report in poultry, previous studies comparing ExPEC strains from humans and companion animals have found ST73 strains causing urinary tract infections in dogs and cats [19, 40], including O6-B2-ST73 clones [41].

Several studies of ST73 clones indicate that this ST is not usually related to any specific type of antimicrobial resistance, unlike others such as the ST131 lineage [1]. Similarly, our results showed that most of these isolates were susceptible to antimicrobials, with only resistance to sulfonamides being commonly reported in 26% (7 isolates), followed by tetracycline in 11% (3 isolates). No multidrug resistance phenotype was observed (Fig 1). Nevertheless, reports on human ST73 have already described multidrug-resistant and ESBL-producing strains [42, 43]. These data indicate the ongoing evolution of this lineage to acquire plasmids carrying different resistance genes.

Our PFGE results divided the clones into three pulsotypes; however only two of the 27 strains showed a reduced similarity (one in group B and one in C, respectively). The 25 strains in group A, although geographically originating from three different states and diverse poultry farms, were all closely related and shared pulsotypes ranging from >90% up to 100%, thus indicating a clonal spread of APEC ST73 in Brazil.

A certain degree of genetic variability within the same ST clones is generally observed irrespective of whether they are human or animal strains, even among those sharing the same geographical locations and sampling periods [17, 44]. The present strains were temporally matched but had very different geographical origins and belonged to diverse farms. Still, the observed divergence was low between strains in group A regardless of the state and farm from where the strains originated. The differences were more pronounced for pulsotypes in groups B and C, thus illustrating varying degrees of genetic polymorphisms within the circulating ST73 population affecting poultry at that time.

In this study, we did not compare human ST73 isolates from the same period. Therefore, further characterization of poultry strains and human isolates will be important to obtain additional data regarding the similarities between avian and human ExPEC ST73.

## Conclusions

In summary, our findings demonstrate close similarities with respect to the serogroup, virulence factors, phylogenetic group, and sequence type of ST73 APEC strains to findings previously reported elsewhere for human ST73 ExPEC. These results provide further information concerning the hypothesis of a connection and the important role of APEC strains associated with human ExPEC infections, either as a source of genetic material supplying virulence genes to other ExPEC or in the transmission of strains. This could be particularly possible for UTIs and bacteremia caused by O6-B2-ST73 isolates given genotypic similarities in virulence between this clonal group and our strains from poultry affected by colibacillosis.

Considering that Brazil is the largest worldwide exporter of broiler meat, our data highlight the importance of surveillance methods for colibacillosis cases affecting the poultry industry while also providing a warning about the zoonotic potential of previously unreported APEC strains from specific clonal groups that have been described as a cause of pandemic ExPEC infections.

## Supporting information

S1 Table Tested virulence factors and references. (DOCX)
