# Supplementary material for: Pandemic extra-intestinal pathogenic Escherichia coli (ExPEC) clonal group O6-B2-ST73 as a cause of avian colibacillosis in Brazil
Source: PLoS One. 2017 Jun 8;12(6):e0178970. doi: 10.1371/journal.pone.0178970 (PMC5464619; doi:10.1371/journal.pone.0178970)
Supplement: S1 Table — (DOCX) [file pone.0178970.s001.docx]

Table S1. Sequence of primer pairs for detection of 27 virulence-related genes.

| Virulence gene | Sequence of primers^1^ (5’-3’) | Reference |
| --- | --- | --- |
| *fimH* | TGCAGAACGGATAAGCCGTGG | [1] |
|  | GCAGTCACCTGCCCTCCGGTA |  |
| *papC* | TGATATCACGCAGTCAGTAGC | [2] |
|  | CCGGCCATATTCACATAAC |  |
| *crl* | TTTCGATTGTCTGGCTGTATG | [3] |
|  | CTTCAGATTCAGCGTCGTC |  |
| *sfa* | CTCCGGAGAACTGGGTGCATCTTAC | [4] |
|  | CGGAGGAGTAATTACAAACCTGGCA |  |
| *tsh* | GGGAAATGACCTGAATGCTGG | [3] |
|  | CCGCTCATCAGTCAGTACCAC |  |
| *afaBC* | GGCAGAGGGCCGGCAACAGGC | [1] |
|  | CCCGTAACGCGCCAGCATCTC |  |
| *iroN* | AATCCGGCAAAGAGACGAACCGCCT | [5] |
|  | GTTCGGGCAACCCCTGCTTTGACTTT |  |
| *irp2* | AAGGATTCGCTGTTACCGGAC | [2] |
|  | TCGTCGGGCAGCGTTTCTTCT |  |
| *fyuA* | TGATTAACCCCGCGACGGGAA | [1] |
|  | CGCAGTAGGCACGATGTTGTA |  |
| *iutA* | GGCTGGACATCATGGGAACTGG | [5] |
|  | CGTCGGGAACGGGTAGAATCG |  |
| *iucD* | TACCGGATTGTCATATGCAGACCGT | [4] |
|  | AATATCTTCCTCCAGTCCGGAGAAG |  |
| *kps*MT II | GCGCATTTGCTGATACTGTTG | [1] |
|  | CATCCAGACGATAAGCATGAGCA |  |
| *iss* | CAGCAACCCGAACCACTTGATG | [5] |
|  | AGCATTGCCAGAGCGGCAGAA |  |
| *ompT* | TCATCCCGGAAGCCTCCCTCACTACTAT | [5] |
|  | TAGCGTTTGCTGCACTGGCTTCTGATAC |  |
| *cvi/cva* | TCCAAGCGGACCCCTTATAG | [2] |
|  | CGCAGCATAGTTCCATGCT |  |

Table S1. Cont.

| *vat* | TCCTGGGACATAATGGCTAG | [2] |
| --- | --- | --- |
|  | GTGTCAGAACGGAATTGTC |  |
| *sat* | TGCTGGCTCTGGAGGAAC | [2] |
|  | TTGAACATTCAGAGTACCGGG |  |
| *hlyA* | AACAAGGATAAGCACTGTTCTGGCT | [4] |
|  | ACCATATAAGCGGTCATTCCCGTCA |  |
| *cnf*1 | AAGATGGAGTTTCCTATGCAGGAG | [4] |
|  | CATTCAGAGTCCTGCCCTCATTATT |  |
| *astA* | TGCCATCAACACAGTATATCC | [2] |
|  | TAGGATCCTCAGGTCGCGAGTGACGGC |  |
| *usp* | ACATTCACGGCAAGCCTCAG | [6] |
|  | AGCGAGTTCCTGGTGAAAGC |  |
| *ibeA* | TGGAACCCGCTCGTAATATAC | [2] |
|  | CTGCCTGTTCAAGCATTGCA |  |
| *hlyF* | GGCCACAGTCGTTTAGGGTGCTTACC | [5] |
|  | GGCGGTTTAGGCATTCCGATACTCAG |  |
| *malX* (PAI I_CFT073_) | GGACATCCTGTTACAGCGCGCA | [1] |
|  | TCGCCACCAATCACAGCCGAAC |  |
| PAI II_CFT073_ | ATGGATGTTGTATCGCGC | [7] |
|  | ACGAGCATGTGGATCTGC |  |
| PAI I_536_ | TAATGCCGGAGATTCATTGTC | [7] |
|  | AGGATTTGTCTCAGGGCTTT |  |
| PAI II_536_ | CATGTCCAAAGCTCGAGCC | [7] |
|  | CTACGTCAGGCTGGCTTTG |  |

^1^For each virulence gene, the primer sequence at top is forward and at bottom is reverse.

**References**

1. Johnson JR, Stell AL (2000) Extended virulence genotypes of Escherichia coli strains from patients with urosepsis in relation to phylogeny and host compromise. J Infect Dis 181: 261–272.

2. Ewers C, Li G, Wilking H, Kiessling S, Alt K, et al. (2007) Avian pathogenic, uropathogenic, and newborn meningitis-causing Escherichia coli: how closely related are they? Int J Med Microbiol 297: 163–176.

3. Maurer JJ, Brown TP, Steffens WL, Thayer SG (1998) The Occurrence of Ambient Temperature-Regulated Adhesins, Curli, and the Temperature-Sensitive Hemagglutinin Tsh among Avian Escherichia coli. Avian Dis 42: 106.

4. Yamamoto S, Terai A, Yuri K, Kurazono H, Takeda Y, et al. (1995) Detection of urovirulence factors in Escherichia coli by multiplex polymerase chain reaction. FEMS Immunol Med Microbiol 12: 85–90.

5. Johnson TJ, Wannemuehler Y, Doetkott C, Johnson SJ, Rosenberger SC, et al. (2008) Identification of minimal predictors of avian pathogenic Escherichia coli virulence for use as a rapid diagnostic tool. J Clin Microbiol 46: 3987–3996.

6. Bauer RJ, Zhang L, Foxman B, Siitonen A, Jantunen ME, et al. (2002) Molecular epidemiology of 3 putative virulence genes for Escherichia coli urinary tract infection-usp, iha, and iroN(E. coli). J Infect Dis 185: 1521–1524.

7. Sabaté M, Moreno E, Pérez T, Andreu A, Prats G (2006) Pathogenicity island markers in commensal and uropathogenic Escherichia coli isolates. Clin Microbiol Infect 12: 880–886.
